# Effects of honeybee (*Apis cerana*) visiting behaviour on toxic plant (*Tripterygium hypoglaucum*) reproduction

**DOI:** 10.1093/aobpla/plac002

**Published:** 2022-04-14

**Authors:** Shunan Chen, Yunfei Wang, Yi Li, Xuewen Zhang, Jie Wu

**Affiliations:** 1 Institute of Apicultural Research, Chinese Academy of Agricultural Sciences; Key Laboratory of Pollinating Insect Biology, Ministry of Agriculture and Rural Affairs; Key Laboratory of Bee Products for Quality and Safety Control, Ministry of Agriculture and Rural Affairs; Bee Product Quality Supervision and Testing Center, Ministry of Agriculture and Rural Affairs; Beijing 100093, People’s Republic of China; 2 Committee of Communist Youth League, Yunnan Agricultural University, Kunming 650201, People’s Republic of China; 3 Institute of Food Science and Technology, Chinese Academy of Agricultural Sciences, Beijing 100093, People’s Republic of China; 4 Yunnan Academy of Agricultural Sciences, Kunming 661101, People’s Republic of China

**Keywords:** *Apis cerana*, florescence, plant reproduction, toxic nectar, toxic plant, *Tripterygium hypoglaucum*

## Abstract

Honeybees play a significant role in the plant–pollinator interactions of many flowering plants. The ecological and evolutionary consequences of plant–pollinator interactions vary by geographic region, and the effects of honeybees on the reproduction of toxic plants have not been well studied. We measured the florescence of toxic plants, the flower-visiting behaviour of honeybees and the effects of pollination on the fertility, weight and moisture content of seeds. The effects of climatic factors on the number of flowers, and the spatial and temporal variation in pollinator visits were evaluated, and the effects of pollinator visits on seed quality were evaluated. Flower visitors were diverse, climatic factors had a great impact on spatio-temporal flowering variation and the number of bee visits was strongly correlated with the spatio-temporal variation in the number of flowers. Honeybees strongly increase the fullness and weight of seeds. Our study demonstrated a good ecological fit between the spatio-temporal variation in the flowering of toxic plants and the general validity of honeybee pollination syndrome in the south of Hengduan Mountains in East Asia. A linear relationship between honeybee visitation and plant reproduction can benefit the stabilization of plant reproduction.

## Teaser

We measured the florescence of toxic plants (*Tripterygium hypoglaucum*), behaviour of honeybee (*Apis cerana*) flower visiting and the effects of honeybee pollination on the reproduction of toxic plants. Flower visitors were diverse, climate factors had a great impact on the spatio-temporal variation of flowering and the number of bee visits was strongly correlated with the spatio-temporal variation of the number of flowers. Honeybees strongly facilitate the reproduction of toxic plants. Our study demonstrated a high ecological fit between the spatio-temporal variation of *T. hypoglaucum* flowering and the general validity of honeybee pollination syndrome in the south of Hengduan Mountains in East Asia.

## Introduction

Christian Konrad Sprengel (1750–1816) found that plants use nectar as a reward for animals that transport pollen and emphasized that this interaction is important for fruit production and plant reproduction (Waser 2006). Thus, he initiated a new era of research on plant–pollinator interactions (Gottsberger 1996).

Honeybees facilitate cross-pollination for individual plants, promote interactions between plant alleles, induce a variety of genetic combinations, transfer genetic material and enhance the stability of species to generate richer genetic diversity (Genung 2010). The successful reproduction of many wildflowers depends heavily on pollinators, and decades of research have shown that pollinators respond to a variety of flower traits to locate and visit flowers when foraging for floral rewards (Chittka *et al.* 1999; Fenster *et al.* 2004; Endress 2011). Honeybees are important pollinators for many flowering plants. Bees visit flowers to forage nectar and pollen as food. Nectar and pollen provide protein, lipids, vitamins and minerals for worker bees and larvae are considered the most essential nutrient source (Westerkamp 1996). The foraging behaviour of bees affects not only honeybee reproductive success at the colony levels but also plants fertilization success (Zych 2013).

Western honeybees are distributed worldwide and managed for honey production and crop species pollination (Mitchell *et al.* 2017); *Apis cerana* is a native plant–pollinator and another managed species in Asia. Most flowering plants cannot reproduce sexually, and humans would lose many food and other plant products without honeybee pollination. Weather affects honeybee foraging by altering the quantity and quality of food resources (Corbet 1990). Previous studies of plant–pollinator interactions and plant reproductive ecology did not include, or very few, examples from East Asia due to the lack of available data (Johnson *et al.* 2017; Ollerton *et al.* 2017).

*Apis* are presumed to be one of the most important pollinators in East Asia (Ren *et al.* 2018). However, the roles of *Apis* as pollinators in natural ecosystems are still poorly understood because most research on Asian honeybees has been conducted in agricultural ecosystems (Cui and Corlett 2016). Bees feed on nectar from non-toxic plants in most areas in China, but alkaloid-containing nectars attract bees in some areas (Ish-Am and Eisikowitch 1998; Chen *et al.* 2015). The compounds in nectar have been thought to act primarily as deterrents (Gottsberger *et al.* 1984; Inouye and Waller 1984). Alkaloids in *Aconitum* spp. nectar affect the rates of both pollinator visitation and harvest. Thus, these compounds perform the function of defending against nectar thieves but may have co-evolved with nectar availability to maintain the fitness benefits of specialized plant–pollinator relationships (Barlow *et al.* 2017). Toxic plants that attract pollinators may successfully achieve reproduction, but no studies have examined how toxic plants attract bees, how much reward plants offer these bees or whether bees can facilitate plant reproduction. The frequency with which pollinators forage toxic nectar and whether pollinators benefit from plants are unknown.

The thunder god vine, *Tripterygium hypoglaucum*, is a fascinating case because it contains a diterpenoid epoxide, triptolide (TRP), a defensive chemical that is likely noxious to herbivores (Sun *et al.* 2009), but also toxic to bees, including a common Asian honey bee species, *A. cerana* (Tan *et al.* 2007). Feeding caged bees honey candy made from honey derived from bees that foraged *T. hypoglaucum* nectar as well as powdered sugar mixed in a 1:1 mass ratio (resulting in 0.3 µg TRP g^−1^) decreased the survival of the bees (Tan *et al.* 2007).

Both natural and synthetic sources affect sodium channels in honeybees, thus changing the daily behaviour of honeybees, which is related to the dose of compounds in nectar (Oliver *et al.* 2015). At concentrations of 0.5–10 µg TRP mL^−1^, there were no effects of acute exposure on learning. However, memory retention (1 h after the last learning trial) significantly decreased by 56 % following acute consumption of 0.5 µg TRP mL^−1^ (Zhang 2018). Honeybees forage for *T. hypoglaucum* toxic nectar in particular seasons and produce honey that contains toxins from plant components. Honey is poisonous to humans and animals, but the threshold for poisoning and death in humans and other mammals is not known (Chen *et al.* 2015).

*Tripterygium* species are pollinated by insects (Roubik 1995), and although little is known about their pollination biology, their flowers are frequently and regularly visited by honeybees (largely *A. cerana*) (Tan *et al.* 2007), Diptera, solitary wasps and ants when other floral resources are less available.

*Apis cerana* is widely distributed in all regions of Asia. *Apis cerana* can adapt to extreme weather and environmental conditions (Chen *et al.* 2017) and has a long flight duration (Oldroyd and Wongsiri 2006), effective grooming and hygienic behaviour (Peng *et al.* 1987) and cooperative group-level defences (Ono *et al.* 1987). A well-known behaviour of *A. cerana* is aggregation when a colony is exposed to dangers, such as predators or intruders. Guard worker bees produce alarm pheromones that dictate group behaviour (Morse *et al.* 1967; Ono *et al.* 1987, 1995). In addition, *A. cerana* provides considerable economic benefits to the apicultural industry through its high-quality by-products in highland areas, perhaps even more so than *A. mellifera*.

*Apis cerana* is an indigenous bee species in China, and an experiment investigated the effect of bee pollination on toxic plant reproduction in this native habitat. First, we assessed the major pollinators in the natural environment of the Hengduan Mountains. Then, we established domesticated honeybee colonies in this area and observed the florescence of *T. hypoglaucum* and the foraging behaviour of honeybees during flowering. Honeybees were able to freely fly during *T. hypoglaucum* flowering. Our data are the first to provide evidence of the flowering period, the species of insects that visited the flower, the changes in the flower-visiting behaviours of bees and the pollination biology of *T. hypoglaucum*.

## Materials and Methods

### Study sites and design

*Tripterygium hypoglaucum* is a perennial liana distributed in areas with altitudes of 1000–2600 m, and in many concentrated communities in some areas (Chen *et al.* 2015, 2017). This study was conducted in three similarly sized *T. hypoglaucum* samples located in the Hengduan Mountains in the southern region of East Asia. Three locations were identified near 27°39′36″N, 85°5′58″E, within an altitude range of 2000–2200 m. Each patch was 5 m long and 5 m wide and had five plants. The plant space selected for testing in each phytocoenosis was length * width * height = 2 * 2 * 2 m. The vegetation in all three locations was a mixed forest of pines, rhododendrons and eucalyptus. The height of plants was 1–4 m, the branches were densely covered with reddish-brown felt-like hairs and the surface of old perennial branches was glabrous. The leaves of the plants were thin leathery, oblong-ovate, broadly elliptic or narrowly ovate, and their size varied greatly. Inflorescences cymose-paniculate, with more than 50 flowers; the flowers were light yellow and 4–5 mm in diameter (Chinese Flora Editorial Board 1999).

### Florescence

The lowest and highest temperatures at the study sites were recorded during the *T. hypoglaucum* bloom. When flower buds appeared on a plant, the numbers of flower buds, open flowers and withered flowers on three inflorescences were counted at the three test sites and observed continuously for 3 days. After analysing the percentage of flowering, >10 %, >50 % and <10 % of the flowers were classified as being in the blooming-start, blooming-peak and senescence phases, respectively.

### Pollinators

To describe the pattern of bee visits to flowers from 2013 to 2014, we established three natural patches and observed the arrival time and retention time of pollinators within 15 days during blooms. The species and number of visiting insects were monitored daily from 09:00 to 18:00. Bagged flower branches were established to isolate the visitors; flowers were exposed from 19:00 to 06:00 the next morning, but during the remaining time, flower inflorescences were isolated from visitors by a mesh bag. Pollinator visits occurred mainly between 09:00 and 16:00 (Chen *et al.* 2015). Therefore, from May to July 2017, we assessed the major pollinators at the study sites, and no domesticated honeybee colonies were found within 2 km. During *T. hypoglaucum* flowering, we observed diurnal visitors from 09:00 to 18:00 every day and recorded their behaviours for 20 min each time. From 24 to 26 June, and 4 to 6 July 2017, *A. cerana* individuals who visited the flowers from 09:00 to 18:00 daily were observed.

### Pollinator effectiveness

**Figure 1.**
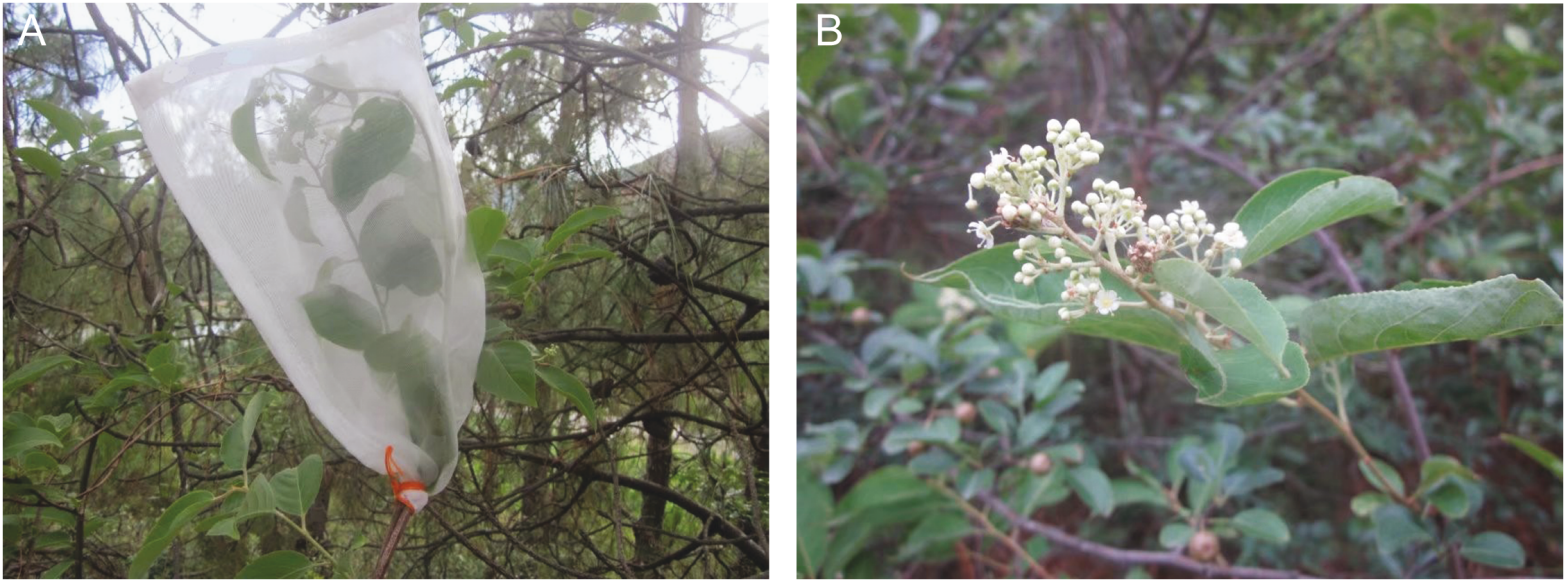
Treated branches and non-treated branches. (A) Bagged flowers to isolate visitors; (B) non-treated branches, which were naturally exposed to visitors. (First author’s photo.).

The effects of pollination by pollinators on plant reproduction were analysed at the three test sites that were more than 2 km from the study sites where major pollinators were assessed. We established three healthy colonies of *A. cerena* that were domesticated in study areas and conducted the experiments. Each colony contained ~15 000 bees. Honeybees were able to freely fly during *T. hypoglaucum* flowering. We randomly selected and bagged flower buds with a sparse net bag 0.1-cm mesh (Fig. 1), and bagged 10 flower branches at each site. The bag was isolated from 21 May to 23 July of 2017 to exclude all flower visitors. As a control, we marked non-treated branches, which were naturally exposed to visitors. At the end of the flowering period, we collected the seeds from flowers that were bagged and those that were visited by insects to compare the differences between the pollinated seeds and the bagged, isolated seeds.

Analysis of the effect of pollination on plants reproduction was conducted at the three selected sites. We collected both pollinated seeds and bagged seeds for examination after flowering. One thousand seeds were randomly selected from among the pollinated seeds and the bagged seeds. The number of full seeds and non-full seeds was recorded, and the percentage (%) of full seeds was calculated. We then weighed 1000 seeds from the two groups using scales and recorded the weight of the seeds. Finally, we put the seeds into a muffle furnace to dry, weighed the seeds again to obtain another weight and calculated the water content of the seeds.

#### Calculation formulas

Seed fullness rate: One thousand seeds were counted, the number of full seeds and non-full seeds was recorded and then the percentage (%) of full seeds was calculated.

$$\begin{array}{l} Seed\;fullness\;rate=the\;number\;of\;seeds\;full/total\;number\;of\;seeds\;*\;\% \end{array}$$

Seed weight: One thousand seeds were randomly selected, and the seeds were weighed using a balance. The average weight was calculated.
Seed moisture content: One thousand seeds were weighed. First, the seeds were weighed to obtain M1. The seeds were placed in a muffle furnace and dried, and their final weight was determined to obtain M2. Finally, the seed moisture content was calculated by determining the difference M1 and M2.

### Statistical analysis

First, we explored the relationship between the florescence of *T. hypoglaucum* and temperature. We calculated the number of buds, flowers and withered flowers, and recorded the change in the lowest temperature and highest temperature during flowering. We also analysed the correlation between the number of flowers and temperature.

To determine the dominant visitor, we recorded the species and number of visitors during flowering. We used ANOVA-LSD to analyse the differences in visits among species, and analysed which pollinator was the dominant pollinator of *T. hypoglaucum*. In addition, we analysed the visit pattern of the pollinators.

To further study the visit pattern of pollinators, we measured visits under various weather conditions and recorded the temperature. We determined the highest frequency of pollinator visits and the correlation between the pattern of visited flowers and the temperature. We then discussed the effects of temperature on the pollinators. We also discussed the relationship between the pattern of bee visits and the number of flowers.

To analyse the effect of dominant pollinators on plant reproduction, we carried out a comparison between the seeds of pollinated flowers and the seeds of bagged flowers. We calculated the seed fullness rate, the weight of 1000 seeds and the seed moisture content. We used ANOVA-LSD to analyse the differences in pollinated seeds and isolated pollination, and we used Pearson’s L-R *x*^2^ degrees of freedom to analyse the differences between the two group of seeds. We then discussed the effect of pollinators on *T. hypoglaucum* reproduction.

All statistical analyses utilized SPSS, version 18.0.

## Results

### Florescence

**Figure 2.**
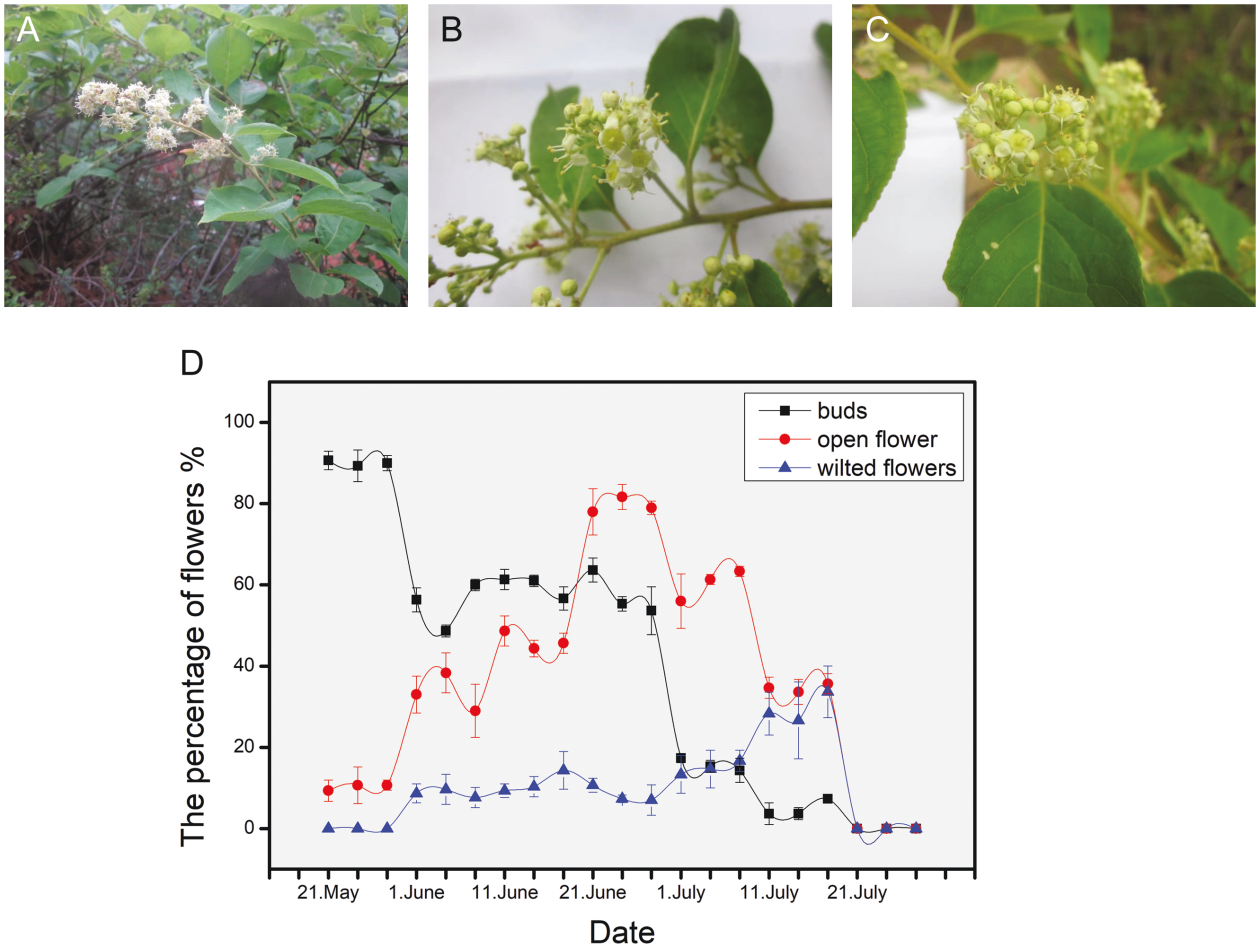
Flowers and florescence of *T. hypoglaucum*. (A and B) Inflorescence; (C) flower; (D) change in the number of buds, flowers and wilted flowers during flowering. The graphs show the mean ± 0.01 standard error. (First author’s photo.).

The florescence of *T. hypoglaucum* lasted from 21 May to 23 July 2017. The blooming-start phase lasted from 21 to 23 May, and the blooming-peak lasted from 13 June to 11 July. The senescence phases began on 11 July (Fig. 2). Flowers secrete a little nectar during each flowering phase. Both temperatures fluctuated in the study sites from May to July. During the experiment, the temperature was measured daily. In May, the highest temperature was 24.71 °C, and the lowest temperature was 14.16 °C. In June, the highest temperature was 25.73 °C, and the lowest temperature was 16.6 °C. In July, the highest temperature was 23.87 °C, and the lowest temperature was 16.16 °C. Other plants did not flower in the same areas as *T. hypoglaucum*.

On 21 May, plants formed many buds and a few flowers. From 1 to 21 June, the number of buds and flowers was increased. The number of flowers was reached in the amount maximum on 23 June, and the number of flowers was increased 6.96 times than 21 May. The number of buds was decreased rapidly after 23 June. From 13 June to 11 July, the number of opened flowers gradually decreased. From 21 July, the number of flowers negatively and finally all faded.

### Visitors

#### Species of visiting insects.

**Figure 3.**
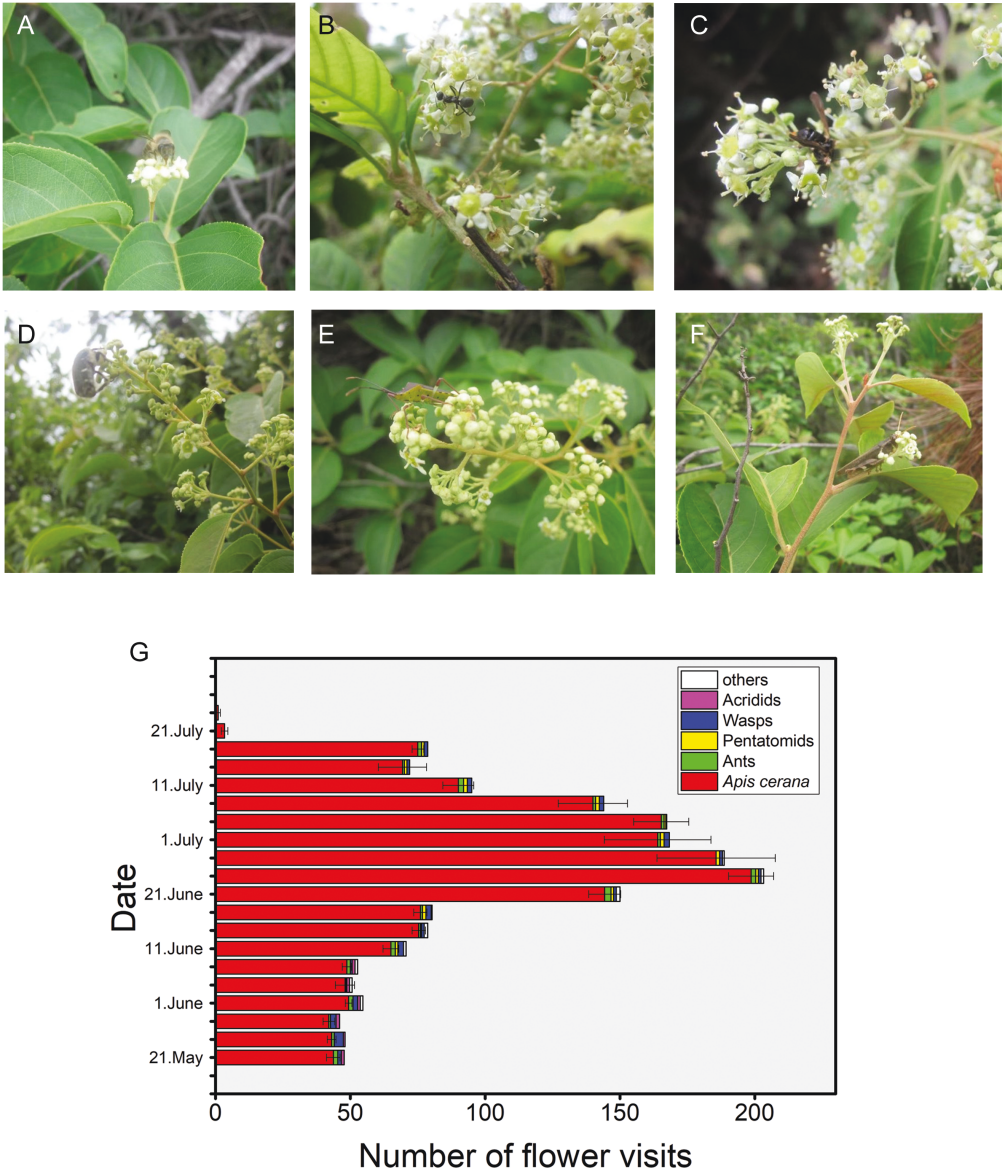
A variety of flower-visiting insects. (A) *Apis cerana*; (B) ants; (C) *Pentatomidae stinkbug*; (D) beetle; (E) wasps; (F) locusts; (G) number of visits by various species. The graphs show the mean ± 1 standard error. (First author’s photo.).

Many diurnal visitors, such as *A. cerana*, ants, *Pentatomidae stinkbug*, wasps and locusts visited the flowers during flowering of *T. hypoglaucum* (Fig. 3). The frequency with which bees visited the flowers was significantly higher than that of other insects (one-way ANOVA-LSD, *F*_5, 377_ = 115.796, *P* < 0.0001). Most bees flew from one flower to another, and the bees occasionally crawled. Honeybees foraged after 09:00 when the temperature was higher, as they were the most frequent species.

#### Patterns of honeybee visiting.

From 21 May to 23 July, honeybees visited the flowers at each phase during flowering. Bees visited the flowers more than any other insects. The visiting behaviour changed across florescence, and the number of daily visits was significantly different (one-way ANOVA-LSD, *F*_20, 42_ = 108.611, *P* < 0.0001). The number of visits gradually increased when the flower bloomed, reached its highest frequency on 22 June and then decreased as the number of flowers decreased. Flower visits by other species were very infrequent.

**Figure 4.**
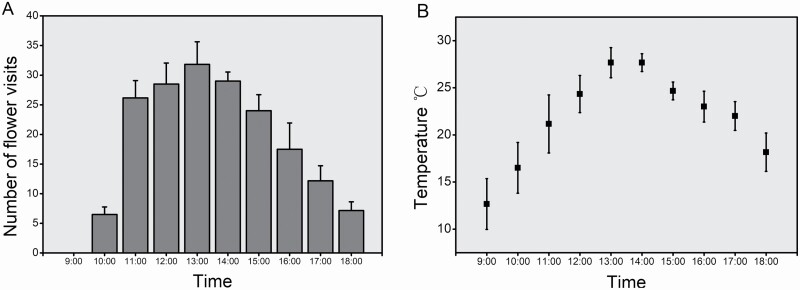
The daily pattern of honeybee visits to flowers, and temperature. (A) The daily pattern of honeybee visits to flowers; (B) the change in temperature during honeybee visits to flower. The graphs show the mean ± 1 (A) and 0.01 (B) standard error.

From 24 to 26 June and 4 to 6 July 2017, we observed honeybees visiting the flowers from 09:00 to 18:00 every day; the frequency of honeybee visits increased from 09:00 to 13:00, and the most frequent visits occurred from 13:00 to 14:00. Before 09:00 to 10:00, the outside temperature was low, and flowers secreted less nectar and pollen. Few insects visited the flowers, and the frequency of honeybee visits from 13:00 to 14:00 was 3.96 times higher than that at 10:00; this number gradually decreased from 13:00 to 18:00. None of the bees foraged after 18:00 (Fig. 4A). The temperature varied greatly from 09:00 to 18:00 (Fig. 4B). The results showed that the frequency of honeybee foraging increased from 09:00 to 13:00, and the highest frequency of flower visits was occurred from 13:00 to 14:00.

The results of analysing the correlation between temperature and honeybee visits showed that visit numbers were significantly correlated with temperature (Pearson’s L-R *N* = 60, *r*_57_ = 0.834, *P* < 0.001). Temperature is an important climatic condition that affects honeybee foraging during flowering.

### Pollinator effectiveness

**Figure 5.**
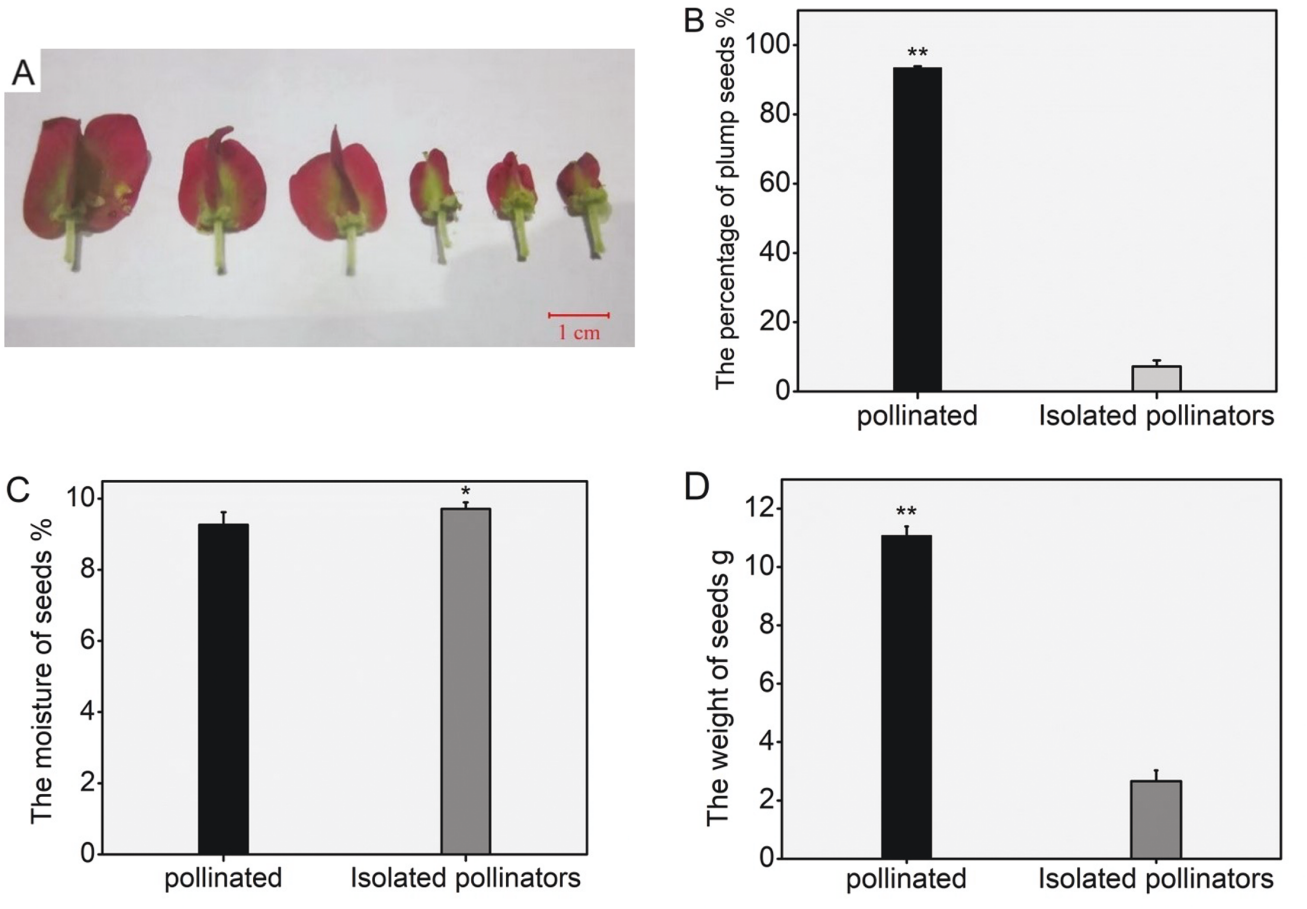
Effects of honeybee pollination on seeds. (A) The first to third fruits from the left were pollinated by bees, and the fourth to sixth fruits were bagged; (B) proportion of plump seeds; (C) seed weight; (D) seed moisture content (*P < 0.05; **P < 0.01). Above each bar, we provide the mean proportion of plump seeds, weight and moisture content and standard error of the seeds pollinated by bees or the seeds from bagged flowers. Stars indicate significant difference between seeds pollinated by bees compared to seeds from bagged flowers (one-way ANOVA-LSD, *P* < 0.05). In total, we tested the proportion of plump seeds of 100 seeds, the moisture content of 100 seeds and the weight of 1000 seeds from three study sites. The graphs show the mean ± 0.01 standard error. (First author’s photo.).

After the artificially controlled pollination experiment, the fruits pollinated by bees were larger than the fruits of bagged flowers (Fig. 5A). The proportion of plump seeds among the pollinated seeds was significantly higher than that isolated seeds (*F*_1, 17_ = 17 984.88, *P* < 0.001; Fig. 5B). The same pattern was also observed in the comparison of seed weight (*F*_1, 17_ = 2441.566, *P* < 0.0001; Fig. 5C). The seed moisture content was lower after pollination by pollinator (*F*_1, 17_ = 10.177, *P* = 0.006; Fig. 5D). *Tripterygium hypoglaucum* successfully attracted bees to visit flowers. Pearson’s L-R *x*^2^ degrees of freedom analysis revealed that bee pollination treatment significantly affected the quality of seeds (Pearson’s L-R *x*^2^_2_ = 223.871, *P* < 0.001).

## Discussion

### Relationship between flowering and temperature

We observed *T. hypoglaucum* flowering from May to July, and other plants in the same region rarely bloom. *Tripterygium hypoglaucum* avoids competition with other floral plants. During the experiment, the highest temperature was 25.73 °C, and the lowest temperature was 14.16 °C. The results of the correlation between the flowering period of *T. hypoglaucum* and the temperature show that the number of buds was significantly correlated with the maximum temperature (Pearson’s L-R *r*_18_ = 0.609, *P* < 0.01). The number of flowers was significantly positively correlated with the minimum temperature (Pearson’s L-R *r*_18_ = 0.784, *P* < 0.01), and the number of wilted flowers was significantly positively correlated with the minimum temperature (Pearson’s L-R *r*_18_ = 0.518, *P* = 0.019). The results suggest that temperature is an important climatic factor affecting the growth and reproduction of *T. hypoglaucum*, but reproductive processes, such as anther development, should be studied in future in-depth research. Anther dehiscence in plant reproductive systems has been shown to be sensitive to abiotic stresses under certain environmental conditions (Franchi *et al.* 2007; Zeng *et al.* 2017). Anther dehiscence is induced by certain combinations of environmental factors, of which temperature is the most important (Franchi *et al.* 2007).

Plants attract foraging honeybees mainly based on their colour and other floral characteristics (Peach 2020). Some plants secrete substances (Baker 1977; Adler 2000), and the chemical constituents of nectar are often closely related to the needs of pollinating insects. Nectar-containing substances usually block some visitors (Adler 2000). Species of the genus *Apis* exhibit a preference for nectar-containing substances (Singaravelan *et al.* 2005). Therefore, some plants secrete nectar containing secondary metabolites and rely entirely on bee pollination (London *et al.* 2003). The characteristics of the flowers and inflorescence of many flowering plants directly influence the pollinator’s selection preferences, and the number of flowers visited by pollinators is positively correlated with the reproduction of plants (Murren and Ellison 1996; O’Connell and Johnston 1998). *Tripterygium hypoglaucum* nectar contains TRP, its flowers are small, and its colour and scent are very light compared with those of non-toxic plants. Compared with the floral plants favoured by pollinators, these flower characteristics are disadvantageous, and are very detrimental for reproduction. Many small flowers form a large inflorescence, which can increase the number of bee visits, increasing the possibility of pollen transmission and the efficiency of pollination.

### Patterns of pollinator visits to flowers

We found that *T. hypoglaucum* species are pollinated by insects, which is consistent with previous studies (Roubik 1995; Tan *et al.* 2007), and their flowers are frequently and regularly visited by honeybees (largely *A. cerana*), Diptera, solitary wasps and ants when other floral resources are less available (Tan *et al.* 2007). In addition, we also found locusts and beetles visiting flowers.

We provide the first evidence describing the patterns of pollinator visits to flowers, and we observed a variety of diurnal insects visiting flowers during *T. hypoglaucum* flowering. Most honeybees fly from one flower to another flower, and honeybees only occasionally crawl. Bees foraged after 09:00 when the temperature was higher, as they were the most frequent species. Whether and how plants can select for the optimal pollinators when plants cannot directly assess floral visitors remains unknown. Although flowers may receive a wide range of visitors, members of only one or two main species act as effective vectors (Schemske and Horvitz 1984; Bawa *et al.* 1985; Bawa 1990). We observed a variety of diurnal insects visiting flowers during the flowering of *T. hypoglaucum*. Our results are similar to those of previous studies (Barlow *et al.* 2017) showing that toxic nectar affects the rates of both pollinator visitation and harvest and that toxins in nectar against nectar thieves. Pollinator visits to flowers were much more frequent than nectar thief visits. Honeybees were the dominate visitors in this study. Insects such as ants, wasps and beetles occasionally visited flowers. Although nectar containing secondary metabolites is considered toxic (Gottsberger *et al.* 1984; Inouye and Waller 1984), bees frequently visited the flowers, suggesting that bees could tolerate toxic nectar, which is also consistent with the characteristics of bees as nectar-euryphagous insects. Bees persistently visited the flowers throughout the flowering period. This is also consistent with the bees’ persistent use of nectar from the same plant. Plants produce floral nectar as a reward for visiting pollinators. Nectar toxins are metabolically expensive for plants to produce and mainly act as a chemical defence against herbivores. The ecological reasons for this are not clear, but it is possible that nectar containing such compounds could be a mechanism underling the specialization of plant–pollinator interactions if insect visitors that are not effective pollinators are susceptible to these compounds (Adler 2000; Tiedeken *et al.* 2014). We suggest that pollinators are selected by *T. hypoglaucum* and that honeybees are the exclusive partners.

### Honeybee foraging behaviour

We observed honeybees visiting flowers at each phase during *T. hypoglaucum* flowering. The number of visits gradually changed with the change in flowering. Honeybee foraging increased with increasing temperature. Temperature is an important climatic condition that affects honeybee foraging. Floral resource availability might be a significant floral-associated factor in determining which flowers bees visit (Harder *et al.* 1994; Johnson 2009). Bees forage according to the amount of food resources provided by plants (Fowler *et al.* 2016). Even in the presence of ideal weather conditions, bees show a low frequency of foraging trips when floral resources are insufficient.

Weather affects the foraging behaviour of bees by altering the quantity and quality of food resources (Corbet 1990; Abou-Shaara *et al.* 2017). Temperature is one of the most important climatic factors that affects honeybee flight (Clarke *et al.* 2018). The foraging behaviour of bees affects not only their colony reproductive success but also the fertilization success of the flowers they visit (Zych *et al.* 2013).

Few studies have assessed the temporal and spatial variability of pollinators during plant flowering. We found that there was a significant positive correlation between the frequency of bee foraging trips and the number of flowers (Pearson’s L-R *N* = 21, *r* = 0.928, *P* < 0.001). The frequency of bee visits increased as the number of flowers increased, and the two patterns were consistent.

It has long been thought that plants that bloom at the same time in nature exhibit significant competition among species for attracting pollinators. However, a growing number of studies have found that floral plant species interact with each other to promote reproduction during the same flowering period (Feinsinger *et al.* 1986, 1987). There is a ‘magnet species effect’ that occurs among these plant species. A flowering plant whose flowers secrete nectar and pollen can promote the pollination success of the flowers of adjacent species, which cannot secrete nectar and pollen or secrete less nectar and pollen (Thomson 1978; Ferdy *et al.* 1998; Juillet *et al.* 2007). Toxic nectar may serve as a filter against ineffective pollinators (Masters 1991), while toxic nectar can, in this context, preserve nectar for legitimate pollinators (Masters 1991; Gosselin *et al.* 2013; Nicolson *et al.* 2015; Thomson *et al.* 2015). Toxins in nectar are probably a strategy developed by *T. hypoglaucum* to reduce the cost of pollination during reproduction, and attract the most effective pollinator.

### The effect of pollinators on seeds

We provide the first data about *T. hypoglaucum* species pollination biology, *T. hypoglaucum* successfully attracted bees to visit the flowers. Honeybee pollination significantly affected the quality of *T. hypoglaucum* seeds. Pollinators visit different individuals in the same plant species, promoting interaction of plant alleles, inducing a variety of genetic combinations, transferring genetic material and enhancing the stability of species to generate richer genetic diversity (Ramanatha and Hodgkin 2002; Genung *et al.* 2010). Moreover, studies have shown that pollinators such as bees promote plant species reproduction (Panda *et al.* 1988; Mahfouz *et al.* 2012) and improve seed quality and yield (Barriault *et al.* 2009).

Few studies have assessed the effect of spatial and temporal changes in pollinators on seeds. We found the fertility and weight of seeds pollinated by bees significantly increased, and the seed quality significantly improved. Honeybees are effective pollinators that facilitate the reproduction of *T. hypoglaucum*, and honeybee pollination can improve seed quality. However, a more precise answer will require more longer-term studies.

## Conclusions

The present study found that toxic *T. hypoglaucum* blooms occurred from May to July, while other plants in the same area rarely bloom. *Tripterygium hypoglaucum* avoids competition with other florescent plants. We observed the various insects that visited the flowers during flowering. Bees were the main pollinators. Honeybee pollination significantly affected the quality of seeds. However, a more exact understanding of the interaction between *T. hypoglaucum* and bees requires further research. The results are useful for understanding the co-evolution of plant nectar metabolites and pollinators and for the management of *A. cerena*, which is now widely reared throughout Asia.

## Supporting Information

The following additional information is available in the online version of this article—

**Table S1.** Temperature.

**Table S2.** Change in the number of buds, flowers and wilted flowers during flowering.

**Table S3.** The number of flower visits by variety insects.

**Table S4.** The daily pattern of honeybees visits to flowers, and the change in temperature during honeybees visits to flower.

**Table S5.** Proportion of plump seeds, seed weight and seed moisture content pollinated by bees and without pollinator.

plac002_suppl_Supplementary_Data

## Data Availability

Date traits used for analysis in this publication can be found in the **Supporting Information**.

## Notes

Plant–Animal Interactions, Pollination Ecology, Reproductive Biology. Chief Editor: F. Xavier Picó
