# Supplementary material for: Effects of honeybee (Apis cerana) visiting behaviour on toxic plant (Tripterygium hypoglaucum) reproduction
Source: AoB Plants. 2022 Apr 14;14(3):plac002. doi: 10.1093/aobpla/plac002 (PMC9071085; doi:10.1093/aobpla/plac002)
Supplement: plac002_suppl_Supplementary_Data [file plac002_suppl_supplementary_data.pdf]

Supplementary Tables S1 to S5

Table S1 Temperature

Date	maximum temperature ℃	minimum temperature ℃
1.May	25	14
2.May	26	15
3.May	26	11
4.May	27	13
5.May	26	11
6.May	25	13
7.May	27	13
8.May	28	14
9.May	29	15
10.May	29	15
11.May	27	14
12.May	23	14
13.May	26	16
14.May	24	12
15.May	16	11
16.May	16	10
17.May	17	11
18.May	22	15
19.May	25	16
20.May	27	16
21.May	28	16
22.May	29	16
23.May	29	17
24.May	28	15
25.May	24	14
26.May	20	13
27.May	21	13
28.May	23	15
29.May	23	17
30.May	24	16
31.May	26	18
1.June	25	17
2.June	24	16
3.June	25	17
4.June	24	15
5.June	27	16
6.June	28	15
7.June	28	16

8.June	28	17
9.June	29	18
10.June	29	18
11.June	27	18
12.June	27	18
13.June	27	18
14.June	27	18
15.June	27	18
16.June	27	18
17.June	27	18
18.June	27	18
19.June	25	17
20.June	25	17
21.June	26	18
22.June	25	18
23.June	26	17
24.June	25	18
25.June	25	17
26.June	27	17
27.June	21	17
28.June	18	0
29.June	25	18
30.June	21	15
1.July	22	16
2.July	21	17
3.July	22	16
4.July	21	17
5.July	25	18
6.July	27	18
7.July	26	19
8.July	24	17
9.July	23	14
10.July	19	14
11.July	25	15
12.July	25	15
13.July	21	15
14.July	25	16
15.July	24	16
16.July	23	15
17.July	25	16
18.July	24	16
19.July	23	15
20.July	26	16

21.July	21	15
22.July	21	15
23.July	23	16
24.July	24	17
25.July	23	17
26.July	25	16
27.July	25	16
28.July	26	16
29.July	27	17
30.July	27	17
31.July	27	18

Table S2 Change in the number of buds, flowers and wilted flowers during flowering

21 May.2017					
Sampling point	Number of collected samples	buds(n)	open flowers (n)	wiletd flowers	
A	100	93	7	0	
B	100	87	13	0	
C	100	92	8	0	
22 May.2017					
Sampling point	Number of collected samples	buds(n)	open flowers (n)	wiletd flowers	
A	100	95	5	0	
B	100	84	16	0	
C	100	89	11	0	
23 May.2017					
Sampling point	Number of collected samples	buds(n)	open flowers (n)	wiletd flowers	
A	100	89	11	0	
B	100	88	12	0	
C	100	93	9	0	
1 June.2017					
Sampling point	Number of collected samples	buds(n)	open flowers (n)	wiletd flowers	
A	100	61	27	12	
B	100	53	34	7	
C	100	55	38	7	
2 June.2017					
Sampling point	Number of collected samples	buds(n)	open flowers (n)	wiletd flowers (n)	
A	100	48	32	10	

B	100	47	39	14	
C	100	51	44	5	
3 June.2017					
Sampling point	Number of collected samples	buds(n)	open flowers (n)	wiletd flowers (n)	
A	100	62	21	7	
B	100	60	29	11	
C	100	58	37	5	
11 June.2017					
Sampling point	Number of collected samples	buds(n)	open flowers (n)	wiletd flowers (n)	
A	100	65	44	11	
B	100	61	49	10	
C	100	58	53	7	
12 June.2017					
Sampling point	Number of collected samples	buds(n)	open flowers (n)	wiletd flowers (n)	
A	100	61	42	7	
B	100	59	47	11	
C	100	63	44	13	
13 June.2017					
Sampling point	Number of collected samples	buds(n)	open flowers (n)	wiletd flowers (n)	
A	100	61	43	16	
B	100	56	49	8	
C	100	53	45	19	
21 June.2017					
Sampling point	Number of collected samples	buds(n)	open flowers (n)	wiletd flowers (n)	
A	100	65	75	10	
B	100	59	73	13	
C	100	67	86	9	
22 June.2017					
Sampling point	Number of collected samples	buds(n)	open flowers (n)	wiletd flowers (n)	
A	100	53	79	8	
B	100	58	80	8	
C	100	55	86	6	
23 June.2017					
Sampling point	Number of collected samples	buds(n)	open flowers (n)	wiletd flowers (n)	
A	100	51	77	12	
B	100	63	79	6	

C	100	47	81	3	
1 July.2017					
Sampling point	Number of collected samples	buds(n)	open flowers (n)	wiletd flowers (n)	
A	100	19	63	18	
B	100	17	58	15	
C	100	16	47	7	
2 July.2017					
Sampling point	Number of collected samples	buds(n)	open flowers (n)	wiletd flowers (n)	
A	100	16	61	13	
B	100	17	63	10	
C	100	13	60	21	
3 July.2017					
Sampling point	Number of collected samples	buds(n)	open flowers (n)	wiletd flowers (n)	
A	100	19	62	19	
B	100	13	65	18	
C	100	11	63	13	
11 July.2017					
Sampling point	Number of collected samples	buds(n)	open flowers (n)	wiletd flowers (n)	
A	100	2	36	22	
B	100	1	31	28	
C	100	8	37	35	
12 July.2017					
Sampling point	Number of collected samples	buds(n)	open flowers (n)	wiletd flowers (n)	
A	100	2	31	39	
B	100	3	32	25	
C	100	6	38	16	
13 July.2017					
Sampling point	Number of collected samples	buds(n)	open flowers (n)	wiletd flowers (n)	
A	100	7	33	40	
B	100	9	35	36	
C	100	6	39	25	
21 July.2017					
Sampling point	Number of collected samples	buds(n)	open flowers (n)	wiletd flowers (n)	
A	100	0	0	0	
B	100	0	0	0	
C	100	0	0	0	

22 July.2017					
Sampling point	Number of collected samples	buds(n)	open flowers (n)	wiletd flowers (n)	
A	100	0	0	0	
B	100	0	0	0	
C	100	0	0	0	
23 July.2017					
Sampling point	Number of collected samples	buds(n)	open flowers (n)	wiletd flowers (n)	
A	100	0	0	0	
B	100	0	0	0	
C	100	0	0	0	

Table S3 The number of flower visits by variety insects.

Flower-visiting insects						
Date						
21.May						
Sampling point	*Apis cerana*	Ant	Pentatomid	Wasp	Acridid	Beatles
A	43	1	0	1	1	0
B	41	3	0	1	2	0
C	47	1	0	2	0	0
22.May						
Sampling point	*Apis cerana*	Ant	Pentatomid	Wasp	Acridid	Beatles
A	43	0	0	3	1	0
B	41	2	0	2	0	0
C	45	1	0	5	1	0
23.May						
Sampling point	*Apis cerana*	Ant	Pentatomid	Wasp	Acridid	Beatles
A	40	1	0	2	1	0
B	45	1	0	3	2	0
C	41	0	0	1	1	0
1.June						
Sampling point	*Apis cerana*	Ant	Pentatomid	Wasp	Acridid	Beatles
A	48	3	0	2	1	3
B	51	1	0	1	1	0
C	49	1	0	2	1	0
2.June						
Sampling point	*Apis cerana*	Ant	Pentatomid	Wasp	Acridid	Beatles
A	43	0	0	1	2	2
B	51	1	0	0	1	0

C	50	0	0	0	0	1
3.June						
Sampling point	*Apis cerana*	Ant	Pentatomid	Wasp	Acridid	Beatles
A	51	2	0	1	1	0
B	48	1	0	0	0	2
C	47	1	0	1	2	1
11.June						
Sampling point	*Apis cerana*	Ant	Pentatomid	Wasp	Acridid	Beatles
A	68	5	2	3	0	0
B	66	0	1	1	0	2
C	61	0	0	2	0	1
12.June						
Sampling point	*Apis cerana*	Ant	Pentatomid	Wasp	Acridid	Beatles
A	72	1	1	1	0	1
B	76	1	0	2	0	2
C	78	0	0	0	0	1
13.June						
Sampling point	*Apis cerana*	Ant	Pentatomid	Wasp	Acridid	Beatles
A	73	0	0	1	0	1
B	79	2	4	2	0	0
C	76	0	0	3	0	0
21.June						
Sampling point	*Apis cerana*	Ant	Pentatomid	Wasp	Acridid	Beatles
A	148	6	1	2	0	1
B	136	1	0	1	0	2
C	149	0	2	0	0	1
22.June						
Sampling point	*Apis cerana*	Ant	Pentatomid	Wasp	Acridid	Beatles
A	203	2	1	0	0	0
B	206	3	1	3	0	3
C	187	0	1	0	0	0
23.June						
Sampling point	*Apis cerana*	Ant	Pentatomid	Wasp	Acridid	Beatles
A	205	0	1	2	0	1
B	155	0	3	0	0	0
C	197	0	0	1	0	1
1.July						
Sampling point	*Apis cerana*	Ant	Pentatomid	Wasp	Acridid	Beatles
A	186	3	1	3	0	0
B	138	0	2	2	0	0
C	168	0	1	1	0	0
2.July						
Sampling point	*Apis cerana*	Ant	Pentatomid	Wasp	Acridid	Beatles

A	178	2	0	1	0	0
B	153	1	1	0	0	0
C	165	0	1	0	0	0
3.July						
Sampling point	*Apis cerana*	Ant	Pentatomid	Wasp	Acridid	Beatles
A	158	2	2	2	0	0
B	133	0	1	2	0	0
C	129	1	1	1	0	0
11.July						
Sampling point	*Apis cerana*	Ant	Pentatomid	Wasp	Acridid	Beatles
A	95	3	1	2	0	0
B	93	1	2	1	0	0
C	82	2	1	2	0	0
12.July						
Sampling point	*Apis cerana*	Ant	Pentatomid	Wasp	Acridid	Beatles
A	78	1	2	1	0	0
B	57	1	0	1	0	0
C	73	0	1	1	0	0
13.July						
Sampling point	*Apis cerana*	Ant	Pentatomid	Wasp	Acridid	Beatles
A	77	2	1	2	0	0
B	76	1	1	1	0	0
C	72	1	1	1	0	0
21.July						
Sampling point	*Apis cerana*	Ant	Pentatomid	Wasp	Acridid	Beatles
A	2	0	0	0	0	0
B	3	0	0	0	0	0
C	5	0	0	0	0	0
22.July						
Sampling point	*Apis cerana*	Ant	Pentatomid	Wasp	Acridid	Beatles
A	1	0	0	0	0	0
B	2	0	0	0	0	0
C	0	0	0	0	0	0
23.July						
Sampling point	*Apis cerana*	Ant	Pentatomid	Wasp	Acridid	Beatles
A	0	0	0	0	0	0
B	0	0	0	0	0	0
C	0	0	0	0	0	0

Table S4 The daily pattern of honeybees visits to flowers, and the change in temperature during honeybees visits to flower.

Honeybee visiting flowers	statistics	bees	temperature
24.June			
Time	statistics	bees	temperature
9:00	No.1	0	10℃
	No.2	0	
	No.3	0	
	No.4	0	
	No.5	0	
	No.6	0	
	No.7	0	
	No.8	0	
	No.9	0	
	No.10	0	
	statistics	bees	temperature
10:00	No.1	1	13℃
	No.2	1	
	No.3	1	
	No.4	0	
	No.5	1	
	No.6	2	
	No.7	0	
	No.8	1	
	No.9	0	
	No.10	1	
	statistics	bees	temperature
11:00	No.1	1	18℃
	No.2	2	
	No.3	3	
	No.4	2	
	No.5	2	
	No.6	3	
	No.7	2	
	No.8	3	
	No.9	5	
	No.10	3	
	statistics	bees	temperature
12:00	No.1	3	23℃
	No.2	4	
	No.3	3	
	No.4	3	
	No.5	2	

	No.6	1	
	No.7	0	
	No.8	3	
	No.9	3	
	No.10	2	
	statistics	bees	temperature
13:00	No.1	3	27℃
	No.2	3	
	No.3	2	
	No.4	3	
	No.5	3	
	No.6	2	
	No.7	5	
	No.8	3	
	No.9	2	
	No.10	5	
	statistics	bees	temperature
14:00	No.1	2	28℃
	No.2	3	
	No.3	5	
	No.4	2	
	No.5	3	
	No.6	3	
	No.7	0	
	No.8	5	
	No.9	3	
	No.10	2	
	statistics	bees	temperature
15:00	No.1	3	25℃
	No.2	2	
	No.3	2	
	No.4	3	
	No.5	5	
	No.6	3	
	No.7	3	
	No.8	3	
	No.9	0	
	No.10	1	
	statistics	bees	temperature
16:00	No.1	2	24℃
	No.2	2	
	No.3	3	
	No.4	0	

	No.5	2	
	No.6	1	
	No.7	0	
	No.8	1	
	No.9	0	
	No.10	2	
	statistics	bees	temperature
17:00	No.1	1	22℃
	No.2	0	
	No.3	2	
	No.4	1	
	No.5	3	
	No.6	0	
	No.7	2	
	No.8	0	
	No.9	1	
	No.10	1	
	statistics	bees	temperature
18:00	No.1	0	16℃
	No.2	1	
	No.3	1	
	No.4	0	
	No.5	1	
	No.6	0	
	No.7	1	
	No.8	0	
	No.9	1	
	No.10	1	
25.June			
Time	statistics	bees	temperature
9:00	No.1	0	13℃
	No.2	0	
	No.3	0	
	No.4	0	
	No.5	0	
	No.6	0	
	No.7	0	
	No.8	0	
	No.9	0	
	No.10	0	
	statistics	bees	temperature
10:00	No.1	0	15℃
	No.2	0	

	No.3	1	
	No.4	0	
	No.5	1	
	No.6	1	
	No.7	0	
	No.8	1	
	No.9	0	
	No.10	1	
	statistics	bees	temperature
11:00	No.1	2	18℃
	No.2	2	
	No.3	3	
	No.4	5	
	No.5	3	
	No.6	3	
	No.7	2	
	No.8	5	
	No.9	3	
	No.10	2	
	statistics	bees	temperature
12:00	No.1	3	23℃
	No.2	5	
	No.3	3	
	No.4	3	
	No.5	2	
	No.6	1	
	No.7	5	
	No.8	2	
	No.9	6	
	No.10	3	
	statistics	bees	temperature
13:00	No.1	3	27℃
	No.2	5	
	No.3	3	
	No.4	6	
	No.5	3	
	No.6	2	
	No.7	3	
	No.8	5	
	No.9	3	
	No.10	5	
	statistics	bees	temperature
14:00	No.1	3	28℃

	No.2	5	
	No.3	2	
	No.4	3	
	No.5	2	
	No.6	5	
	No.7	1	
	No.8	2	
	No.9	5	
	No.10	3	
	statistics	bees	temperature
15:00	No.1	2	23℃
	No.2	1	
	No.3	3	
	No.4	5	
	No.5	3	
	No.6	3	
	No.7	2	
	No.8	2	
	No.9	3	
	No.10	3	
	statistics	bees	temperature
16:00	No.1	3	21℃
	No.2	2	
	No.3	0	
	No.4	3	
	No.5	4	
	No.6	3	
	No.7	2	
	No.8	0	
	No.9	3	
	No.10	3	
	statistics	bees	temperature
17:00	No.1	1	21℃
	No.2	2	
	No.3	1	
	No.4	1	
	No.5	3	
	No.6	0	
	No.7	2	
	No.8	2	
	No.9	3	
	No.10	2	
	statistics	bees	temperature

18:00	No.1	0	18℃
	No.2	0	
	No.3	1	
	No.4	0	
	No.5	1	
	No.6	2	
	No.7	1	
	No.8	2	
	No.9	1	
	No.10	2	
26.June			
Time	statistics	bees	temperature
9:00	No.1	0	9℃
	No.2	0	
	No.3	0	
	No.4	0	
	No.5	0	
	No.6	0	
	No.7	0	
	No.8	0	
	No.9	0	
	No.10	0	
	statistics	bees	temperature
10:00	No.1	0	16℃
	No.2	1	
	No.3	1	
	No.4	0	
	No.5	1	
	No.6	2	
	No.7	0	
	No.8	1	
	No.9	0	
	No.10	1	
	statistics	bees	temperature
11:00	No.1	2	19℃
	No.2	3	
	No.3	4	
	No.4	3	
	No.5	3	
	No.6	3	
	No.7	4	
	No.8	2	
	No.9	3	

	No.10	2	
	statistics	bees	temperature
12:00	No.1	5	22℃
	No.2	4	
	No.3	5	
	No.4	1	
	No.5	5	
	No.6	2	
	No.7	3	
	No.8	1	
	No.9	3	
	No.10	1	
	statistics	bees	temperature
13:00	No.1	5	25℃
	No.2	6	
	No.3	3	
	No.4	2	
	No.5	5	
	No.6	2	
	No.7	5	
	No.8	3	
	No.9	1	
	No.10	3	
	statistics	bees	temperature
14:00	No.1	2	26℃
	No.2	1	
	No.3	4	
	No.4	5	
	No.5	3	
	No.6	2	
	No.7	3	
	No.8	3	
	No.9	3	
	No.10	2	
	statistics	bees	temperature
15:00	No.1	3	25℃
	No.2	3	
	No.3	3	
	No.4	2	
	No.5	2	
	No.6	0	
	No.7	3	
	No.8	3	

	No.9	1	
	No.10	1	
	statistics	bees	temperature
16:00	No.1	1	22℃
	No.2	2	
	No.3	1	
	No.4	2	
	No.5	1	
	No.6	2	
	No.7	3	
	No.8	0	
	No.9	3	
	No.10	1	
	statistics	bees	temperature
17:00	No.1	0	20℃
	No.2	3	
	No.3	0	
	No.4	1	
	No.5	2	
	No.6	1	
	No.7	2	
	No.8	5	
	No.9	0	
	No.10	0	
	statistics	bees	temperature
18:00	No.1	0	16℃
	No.2	0	
	No.3	0	
	No.4	1	
	No.5	2	
	No.6	0	
	No.7	1	
	No.8	1	
	No.9	2	
	No.10	1	
4.July			
Time	statistics	bees	temperature
9:00	No.1	0	12℃
	No.2	0	
	No.3	0	
	No.4	0	
	No.5	0	
	No.6	0	

	No.7	0	
	No.8	0	
	No.9	0	
	No.10	0	
	statistics	bees	temperature
10:00	No.1	0	15℃
	No.2	3	
	No.3	0	
	No.4	0	
	No.5	2	
	No.6	2	
	No.7	0	
	No.8	0	
	No.9	1	
	No.10	0	
	statistics	bees	temperature
11:00	No.1	0	22℃
	No.2	2	
	No.3	1	
	No.4	3	
	No.5	2	
	No.6	5	
	No.7	0	
	No.8	1	
	No.9	7	
	No.10	5	
	statistics	bees	temperature
12:00	No.1	5	25℃
	No.2	2	
	No.3	1	
	No.4	5	
	No.5	0	
	No.6	1	
	No.7	0	
	No.8	4	
	No.9	5	
	No.10	1	
	statistics	bees	temperature
13:00	No.1	2	29℃
	No.2	3	
	No.3	2	
	No.4	4	
	No.5	11	

	No.6	2	
	No.7	1	
	No.8	2	
	No.9	1	
	No.10	2	
	statistics	bees	temperature
14:00	No.1	1	28℃
	No.2	6	
	No.3	3	
	No.4	0	
	No.5	3	
	No.6	5	
	No.7	1	
	No.8	2	
	No.9	7	
	No.10	1	
	statistics	bees	temperature
15:00	No.1	2	26℃
	No.2	2	
	No.3	1	
	No.4	1	
	No.5	7	
	No.6	1	
	No.7	4	
	No.8	1	
	No.9	2	
	No.10	3	
	statistics	bees	temperature
16:00	No.1	0	22℃
	No.2	2	
	No.3	1	
	No.4	6	
	No.5	2	
	No.6	0	
	No.7	1	
	No.8	0	
	No.9	1	
	No.10	0	
	statistics	bees	temperature
17:00	No.1	1	22℃
	No.2	1	
	No.3	1	
	No.4	1	

	No.5	1	
	No.6	1	
	No.7	1	
	No.8	1	
	No.9	0	
	No.10	2	
	statistics	bees	temperature
18:00	No.1	1	18℃
	No.2	1	
	No.3	0	
	No.4	0	
	No.5	1	
	No.6	0	
	No.7	1	
	No.8	0	
	No.9	0	
	No.10	2	
5.July			
Time	statistics	bees	temperature
9:00	No.1	0	16℃
	No.2	0	
	No.3	0	
	No.4	0	
	No.5	0	
	No.6	0	
	No.7	0	
	No.8	0	
	No.9	0	
	No.10	0	
	statistics	bees	temperature
10:00	No.1	0	19℃
	No.2	0	
	No.3	0	
	No.4	0	
	No.5	0	
	No.6	3	
	No.7	2	
	No.8	0	
	No.9	0	
	No.10	0	
	statistics	bees	temperature
11:00	No.1	0	26℃
	No.2	1	

	No.3	1	
	No.4	7	
	No.5	4	
	No.6	5	
	No.7	2	
	No.8	7	
	No.9	1	
	No.10	2	
	statistics	bees	temperature
12:00	No.1	9	28℃
	No.2	1	
	No.3	1	
	No.4	1	
	No.5	2	
	No.6	7	
	No.7	5	
	No.8	4	
	No.9	1	
	No.10	1	
	statistics	bees	temperature
13:00	No.1	1	30℃
	No.2	3	
	No.3	3	
	No.4	6	
	No.5	3	
	No.6	0	
	No.7	5	
	No.8	1	
	No.9	7	
	No.10	2	
	statistics	bees	temperature
14:00	No.1	1	27℃
	No.2	2	
	No.3	4	
	No.4	3	
	No.5	5	
	No.6	5	
	No.7	1	
	No.8	4	
	No.9	4	
	No.10	2	
	statistics	bees	temperature
15:00	No.1	0	25℃

	No.2	0	
	No.3	3	
	No.4	2	
	No.5	4	
	No.6	4	
	No.7	5	
	No.8	4	
	No.9	4	
	No.10	1	
	statistics	bees	temperature
16:00	No.1	9	26℃
	No.2	0	
	No.3	1	
	No.4	2	
	No.5	1	
	No.6	1	
	No.7	2	
	No.8	1	
	No.9	3	
	No.10	4	
	statistics	bees	temperature
17:00	No.1	0	25℃
	No.2	1	
	No.3	0	
	No.4	1	
	No.5	0	
	No.6	1	
	No.7	2	
	No.8	2	
	No.9	1	
	No.10	3	
	statistics	bees	temperature
18:00	No.1	1	22℃
	No.2	1	
	No.3	0	
	No.4	0	
	No.5	0	
	No.6	0	
	No.7	0	
	No.8	1	
	No.9	2	
	No.10	2	
6.July			

Time	statistics	bees	temperature
9:00	No.1	0	16℃
	No.2	0	
	No.3	0	
	No.4	0	
	No.5	0	
	No.6	0	
	No.7	0	
	No.8	0	
	No.9	0	
	No.10	0	
	statistics	bees	temperature
10:00	No.1	3	21℃
	No.2	1	
	No.3	1	
	No.4	0	
	No.5	0	
	No.6	0	
	No.7	0	
	No.8	1	
	No.9	0	
	No.10	0	
	statistics	bees	temperature
11:00	No.1	0	24℃
	No.2	3	
	No.3	2	
	No.4	1	
	No.5	3	
	No.6	5	
	No.7	1	
	No.8	5	
	No.9	9	
	No.10	1	
	statistics	bees	temperature
12:00	No.1	1	25℃
	No.2	8	
	No.3	2	
	No.4	1	
	No.5	8	
	No.6	2	
	No.7	2	
	No.8	1	
	No.9	2	

	No.10	1	
	statistics	bees	temperature
13:00	No.1	15	28℃
	No.2	0	
	No.3	1	
	No.4	1	
	No.5	2	
	No.6	2	
	No.7	1	
	No.8	2	
	No.9	1	
	No.10	1	
	statistics	bees	temperature
14:00	No.1	6	29℃
	No.2	0	
	No.3	0	
	No.4	8	
	No.5	1	
	No.6	4	
	No.7	0	
	No.8	6	
	No.9	1	
	No.10	1	
	statistics	bees	temperature
15:00	No.1	1	24℃
	No.2	3	
	No.3	2	
	No.4	2	
	No.5	4	
	No.6	1	
	No.7	5	
	No.8	1	
	No.9	0	
	No.10	1	
	statistics	bees	temperature
16:00	No.1	3	23℃
	No.2	1	
	No.3	1	
	No.4	0	
	No.5	2	
	No.6	4	
	No.7	1	
	No.8	1	

	No.9	1	
	No.10	2	
	statistics	bees	temperature
17:00	No.1	1	22℃
	No.2	2	
	No.3	0	
	No.4	1	
	No.5	0	
	No.6	1	
	No.7	1	
	No.8	2	
	No.9	1	
	No.10	1	
	statistics	bees	temperature
18:00	No.1	0	19℃
	No.2	0	
	No.3	0	
	No.4	1	
	No.5	1	
	No.6	0	
	No.7	1	
	No.8	1	
	No.9	1	
	No.10	1	

Table S5 Proportion of plump seeds, seed weight and seed moisture content pollinated by bees and

without pollinator.

Seeds	test point			
pollinating		plump seed (%)	seed moisture content (%)	seed weight (g)
	A	96.3	8.86	11.656
		95.6	8.79	11.312
		93.7	8.83	11.516
	B	91.62	9.58	10.837
		91.35	9.67	10.889
		90.83	9.79	10.795
	C	94.02	9.23	10.968
		93.97	9.33	10.836
		93.86	9.29	10.891
bagged flower		plump seed (%)	seed moisture content (%)	seed weight (g)
	A	7.61	9.35	2.031
		6.12	9.61	2.673
		7.63	9.52	2.626
	B	7.32	9.63	3.036
		7.37	9.83	2.851
		7.06	9.88	2.562
	C	7.16	9.89	3.06
		7.25	9.86	3.03
		7.33	9.83	2.07
